# Cost-effectiveness of expanding access to intrauterine device provision by nurses in the public health system: an economic decision analysis using a Markov model, Pernambuco, 2023

**DOI:** 10.1590/S2237-96222025v34e20240803.en

**Published:** 2026-02-23

**Authors:** Juliana da Silva Nogueira, Camilla Maria Ferreira de Aquino, Juliana Gonçalves de Araújo, Noemia Teixeira de Siqueira, Umbelina Cravo Teixeira Lagioia, Agostinho de Sousa Machado, Adriana Falangola Benjamin Bezerra

**Affiliations:** 1 Universidade Federal de Pernambuco, Ambulatório de Ginecologia e Obstetrícia, Recife, PE, Brazil; 2 Instituto Federal de Pernambuco, Departamento de Enfermagem, Recife, PE, Brazil; 3 Universidade de Pernambuco, Coordenação do MBA em Gestão Empresarial, Recife, PE, Brazil; 4 University of York, York, NYorks, England; 5 Universidade Federal de Pernambuco, Departamento de Ciências Contábeis, Recife, PE, Brazil; 6 Universidade Federal de Pernambuco, Departamento de Ginecologia, Recife, PE, Brazil; 7 Universidade Federal de Pernambuco, Departamento de Saúde Pública, Recife, PE, Brazil

**Keywords:** Nurses, Professional Training, Effective Access to Health Services, Health Services Needs and Demands, Health Evaluation, Enfermeras y Enfermeros, Capacitación Profesional, Acceso Efectivo a los Servicios de Salud, Necesidades y Demandas de Servicios de Salud, Evaluación en Salud

## Abstract

**Objective:**

To analyze the cost-effectiveness of increasing the provision and insertion of intrauterine devices following the training of nurses.

**Methods:**

An economic decision analysis using a Markov model was conducted in TreeAge Pro Healthcare 2023 to compare four scenarios in Pernambuco, based on the 2023 population estimate. The current scenario (reference) has a 0.24% provision rate of intrauterine devices. The other scenarios projected increases to 2% (Scenario 1), 3.2% (Scenario 2), and 4% (Scenario 3). Costs related to training and to the distribution of intrauterine devices were analyzed using the absorption costing method. Utilities were estimated based on the literature, adopting the Brazilian willingness-to-pay threshold of up to BRL 40,000.00, allowing flexibility up to BRL 120,000.00 per quality-adjusted life year (QALY).

**Results:**

Scenario 1 (2% provision) showed the best cost-effectiveness ratio compared to the current scenario, with an incremental cost of BRL 73,720.20 per QALY gained and benefits observed three months after training. The other scenarios were dominated, meaning that they presented higher costs and lower effectiveness, making them disadvantageous in the short term.

**Conclusion:**

Expanding the provision to 2% through nurse training is a cost-effective strategy compared to the current level. In addition to optimizing public resources, this measure may reduce the unmet demand for contraception.

## Resumen

**Objetivo:**

Analizar el costo-efectividad del aumento de la oferta y de la inserción de dispositivos intrauterinos tras la capacitación de enfermeros.

**Métodos:**

Se utilizó un análisis de decisión económica con modelo de Markov en el software TreeAge Pro Healthcare 2023 para comparar cuatro escenarios en Pernambuco, considerando la población estimada para 2023. El escenario actual (de referencia) presenta una oferta del 0,24% de dispositivos intrauterinos. Los demás escenarios proyectaron incrementos a 2% (Escenario 1), 3,2% (Escenario 2) y 4% (Escenario 3). Se analizaron los costos de capacitación y de distribución de los dispositivos intrauterinos, empleando el método de costeo por absorción. Las utilidades se estimaron con base en la literatura, adoptando el umbral brasileño de disposición a pagar de hasta R$ 40.000,00, con flexibilidad admitida hasta R$ 120.000,00 por año de vida ajustado por calidad.

**Resultados:**

El Escenario 1 (2% de oferta) presentó la mejor relación de costo-efectividad en comparación con el escenario actual, con un costo incremental de R$ 73.720,20 por año de vida ajustado por calidad, con ganancias observadas tras tres meses de capacitación. Los demás escenarios fueron dominados, es decir, mostraron aumento de costos y pérdida de efectividad, resultando desventajosos a corto plazo.

**Conclusión:**

Ampliar la oferta al 2% mediante la capacitación de enfermeros es una estrategia costo-efectiva en comparación con la oferta actual. Además de optimizar los recursos públicos, esta medida puede reducir la demanda insatisfecha de anticoncepción.

**Ethical aspects**

This research respected ethical principles, having obtained the following approval data:

Research ethics committee: Hospital das Clínicas de Pernambuco

Opinion number: 6,338,193

Approval date: 2/10/2023

Certificate of submission for ethical appraisal: 74238023.5.0000.8807

Informed consent form: Exempt.

## Introduction 

Reproductive planning contributes to reducing unintended pregnancies and aligns with the United Nations Sustainable Development Goals (1). In Brazil, both short- and long-acting contraceptive methods are provided free of charge. However, adherence to long-acting reversible contraceptives remains low, with a prevalence of only 2% (2). The population has greater access to short-acting hormonal methods, which generally have a higher failure rate than intrauterine devices. This low adherence contributes to high rates of unplanned pregnancies, abortions, and maternal morbidity (3,4).

The high rate of adolescent pregnancy in Brazil generates social and economic impacts that exceed the cost of expanding access to these contraceptives (5). To improve access, it is not enough for the Brazilian Unified Health System (*Sistema Único de Saúde*, SUS) to simply acquire these supplies; it is also necessary to train professionals to perform the insertions (6). Currently, physicians predominate in the insertion of these devices; however, in the Northeast, nurses have performed 47% of the procedures, indicating a trend toward greater participation by nursing professionals (7).

Data from the Department of Informatics of the Brazilian Unified Health System (*Departamento de Informática do Sistema Único de Saúde*, DATASUS) indicate growth in the provision of intrauterine devices in Pernambuco—from 870 insertions in 2020 to 13,417 in 2022. Use within the public health system rose from 0.029% in 2020 to 0.24% in 2022. However, in 2017, 44% of the 24,047 distributed devices expired without records of use, resulting in a loss of BRL 1,011,180.00 (USD 204,916.30). In 2024, there were 31,195 units, totaling BRL 2,963,525.00 (USD 553,164.78), which needed to be used by 2028 to prevent further losses. 

Despite the availability of supplies to offer long-acting contraceptive methods (8), access to sexual and reproductive health services in Brazil remains fragmented and unequal, as there is an insufficient number of trained physicians and nurses to maintain supply proportional to demand (9). In 2022, the prevalence of unintended pregnancy was 62%, with a higher incidence among young, Brwon (Brazilian mixed-race) women without partners (2), reinforcing the need for more effective strategies in contraceptive provision. Training nurses can expand access. 

Resolution No. 690/2022 of the Federal Nursing Council (*Conselho Federal de Enfermagem*, Cofen) established guidelines for training, allowing nurses to conduct consultations and insert intrauterine devices (10). Since 2022, the Federal and Regional Nursing Councils have initiated training programs and the systematization of procedures across Brazilian states through the Gynecological Nursing Consultation Program, emphasizing sexual and reproductive planning (11). Adequate training enables nurses to provide health education, raise user awareness, and deliver comprehensive, safe, and effective care (12,13). Insertion of this type of contraceptive is simple, with a low incidence of severe pain and a low risk of complications (14). International experiences, such as in Canada, have shown that expanding the scope of nursing practice can overcome barriers to accessing reproductive health care (15). 

Given the potential economic impacts of expanding the number of trained professionals—along with infrastructure adjustments, supply acquisition, and reorganization of health service routines—this intervention should first be evaluated in terms of its cost-effectiveness, as recommended by the National Commission for the Incorporation of Technologies in the Brazilian Unified Health System (*Comissão Nacional de Incorporação de Tecnologias no Sistema Único de Saúde*, CONITEC) (16).

In light of this, the present study aimed to analyze the cost-effectiveness of increasing the provision and insertion of intrauterine devices following nurse training. The results aim to provide evidence to support public policies that promote effective contraceptive methods, thereby improving the health and economic indicators related to Brazilian women.

## Methods 

### Study design

A cost-effectiveness evaluation was conducted of expanding intrauterine device provision by nurses within the public health network of Pernambuco. The model development followed the 2022 Consolidated Health Economic Evaluation Reporting Standards (CHEERS) guidelines and was conducted using TreeAge Pro Healthcare 2023.

### Target population 

The study participants were sexually active girls and women aged 10 to 49 years who did not wish to become pregnant and were attended by nurses after receiving training.

The sample for each scenario was based on data from the Outpatient Information System (*Sistema de Informação Ambulatorial*, SIA) of the Department of Informatics of the Brazilian Unified Health System (DATASUS), considering the number of intrauterine device insertions in Pernambuco in 2023. Sample size was calculated based on the prevalence of intrauterine device use in the hypothetical scenarios, with N*0.0024=600 for the Pernambuco (PE) scenario, N*0.02=734 for Scenario 1, N*0.032=1,226 for Scenario 2, and N*0.04=1,532 for Scenario 3.

### Context and setting

In Pernambuco, intrauterine device insertions may take place in primary health care services managed by municipal authorities, such as Health Centers and Polyclinics. At the state level, regional hospitals include reproductive planning outpatient clinics where patients, referred through the 12 regional health divisions, can receive care. For the simulation, 2023 data were used, collected from publicly accessible information systems in Pernambuco, focusing on sociodemographic and primary care data. 

### Study perspective

The primary analytical perspective adopted was that of the Brazilian Unified Health System (SUS). 

### Comparators 

The health technology under study was the insertion of long-acting reversible contraceptive methods by trained primary care nurses. Four scenarios of expanded provision of the same contraceptive method were evaluated: Pernambuco scenario (reference scenario, current provision probability 0.0024); Scenario 1 (increase to 0.02, national average); Scenario 2 (increase to 0.032); and Scenario 3 (increase to 0.04).

### Time horizon

In each scenario, three months after the training sessions was considered. Each training session lasted one month (17), and monthly transitions between health states were adopted, as participants could move from “not pregnant” to “pregnant” during that period. The evaluation period was not extended due to the absence of data on the long-term effects of training on intrauterine device insertions.

### Discount rate

No discount rate was applied because the analysis period was shorter than one year (18).

### Health outcome

Quality-adjusted life years (QALY) were defined as the primary outcome (19). Effectiveness was measured by the ability to achieve desired outcomes, such as pregnancy prevention, through increased provision of intrauterine devices among eligible individuals within the Family Health Strategy (*Estratégia Saúde da Família*, ESF). Statistical and cost-effectiveness analyses were conducted in 2023. 

Utility measures ranged from 0 to 1 and were defined as follows: a) utility with intrauterine device use without pregnancy (value=1); b) utility without intrauterine device use and without pregnancy (value=0.8) (20).

In situations where pregnancy occurred despite intrauterine device use and the desire to avoid pregnancy, reductions were observed in quality of life, perceived well-being, and confidence in the method. For this outcome, decremental utility (disutility) values were assigned to the variable “intrauterine device use with pregnancy,” estimated at –0.1 (20).

### Cost-effectiveness threshold

The willingness-to-pay threshold was set at BRL 40,000.00 per QALY gained. In Brazil, the guidelines for willingness-to-pay thresholds allow flexibility in adopting alternative thresholds when justified by considerations of innovation and equity in health within the SUS context. In such cases, thresholds up to BRL 120,000.00 are considered acceptable (21).

### Costs considered

Direct costs of the strategies under investigation were included, comprising: a) intrauterine device; b) individual team training costs, based on a study conducted in Pernambuco (17); c) cost of unintended pregnancy in Brazil, updated to 2023 (22); and d) cost of individual consultation without intrauterine device insertion (SUS pricing schedule).

### Data sources and measurement 

The technologies evaluated included maintaining the current provision level in Pernambuco (probability=0.0024), calculated from DATASUS data. The hypothetical increase to 0.02 simulated the national probability of intrauterine device use (2). In the second hypothetical scenario, this estimate increased by 60% following the training of primary care professionals for intrauterine device provision and insertion (23), resulting in a probability of 0.032. 

In the last hypothetical scenario, a 100% increase in the nationwide provision was evaluated, corresponding to a 0.04 probability of intrauterine device use. 

The outcomes analyzed included avoided pregnancies and unintended pregnancies with and without copper intrauterine devices. The cost of nurse training was calculated at BRL 1,820.38 per professional, with a unit insertion cost of BRL 91.02 (17). The cost per scenario was the device insertion cost multiplied by the number of eligible users in each scenario. Additional costs, such as consultations without intrauterine device insertion and without unintended pregnancy, were obtained from the SUS price schedule and from Brazilian studies (22,24), adjusted to 2023 using the Extended National Consumer Price Index (*Índice Nacional de Preços ao Consumidor Amplo*, IPCA).

### Markov model

The model was developed to simulate the clinical and economic effects of nurse training for intrauterine device insertion in a hypothetical cohort of women of reproductive age eligible for intrauterine device use, evaluated over three months following the training. The cost-effectiveness evaluation aimed to anticipate the costs and effects of the training and the expanded provision before local health managers implement the intervention. The health technology was tested considering repetitive events (for example: contraceptive method use or nonuse → pregnancy → pregnancy loss → renewed method use or nonuse), with monthly transitional states and time-dependent probabilities and utilities, allowing for a more accurate representation of the clinical structure assessed. Participants could transition among different health states: pregnant without an intrauterine device, not pregnant without an intrauterine device, not pregnant with an intrauterine device, and pregnant with an intrauterine device.

### Sensitivity analysis

Sensitivity analyses were conducted to assess the impact of uncertainty on the results. Cost and utility parameters were included in deterministic (tornado diagram) and probabilistic (Monte Carlo simulation with 10,000 iterations) analyses using data from the literature. The parameters used are presented in Supplementary Table 1.

**Table 1:** Ranking of the cost-effectiveness assessment among the three scenarios of expanded provision of intrauterine device insertion for women who do not wish to become pregnant, according to the incremental cost-effectiveness ratio and net monetary benefits. Pernambuco, 2023 (n=4,092)

Strategy	Dominance		Cost (BRL)	Incremental cost (BRL)	Effectiveness	Incremental effectiveness	Incremental cost-effectiveness ratio	Net monetary benefits
Pernambuco Scenario		Not dominated	3,813	-	4.3	-	-	-
Scenario 1		Not dominated	19,655	15,842	4.5	0.2	73,720.20	233,073,733
Scenario 2		Dominated	48,003	28,348	4.0	-0.5	-55,273.57	206,376,044
Scenario 3		Dominated	73,819	54,165	3.7	-0.7	-73,866.25	194,888,795

## Results 

In the cost-effectiveness analysis, the four scenarios were compared over three months, showing that the Pernambuco and Scenario 1 options were not dominated, while Scenarios 2 and 3 were dominated, meaning they had higher costs without gains in effectiveness. 

Expanding the provision of intrauterine devices from the Pernambuco scenario to Scenario 1 represented a 1,233% increase, calculated by the ratio (0.032–0.0024)/0.0024×100.

The incremental cost-effectiveness ratio also indicated that Scenario 1 is within the alternative willingness-to-pay thresholds, at BRL 73,720.20 per quality-adjusted life year, when values up to BRL 120,000.00 are considered.

Within the cost-effectiveness ranking framework, the assessment of net monetary benefits also confirmed that Scenario 1 is the most cost-effective option, as it is the most advantageous when considering the willingness-to-pay threshold (Table 1). 

**Figure 1:**
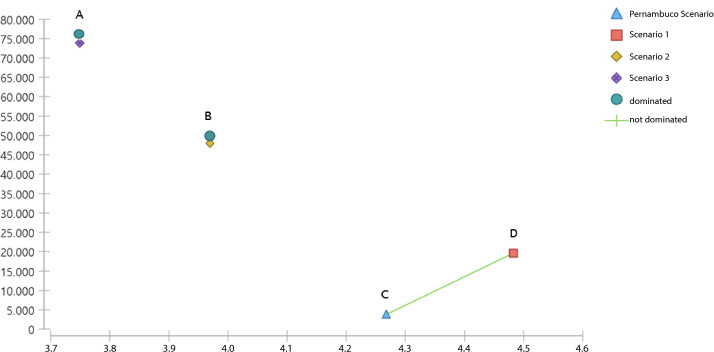
Cost-effectiveness analysis comparing the four scenarios of intrauterine device provision. Pernambuco, 2023 (n=4,092)

A cost-effectiveness acceptability curve was constructed across the three hypothetical scenarios, with Scenario 1 showing the largest incremental gain in effectiveness (QALY gained) at the lowest cost (Figure 1). 

Sensitivity analyses were conducted to assess uncertainty in the model parameters, recalculating costs and utilities (deterministic analysis). 

**Figure 2:**
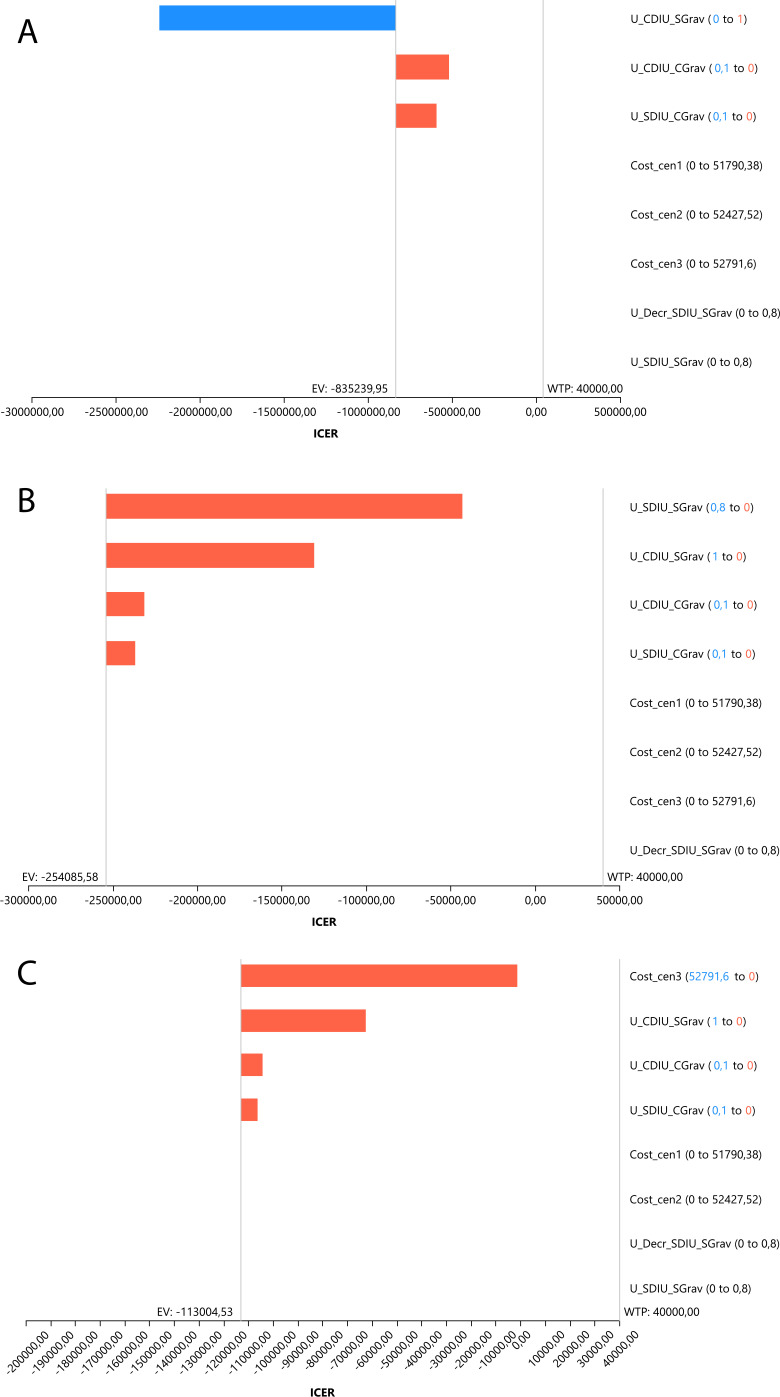
Deterministic sensitivity analysis. Variation in the incremental cost-effectiveness ratio when comparing alternative scenarios (A, B, and C) with the Pernambuco scenario, at a willingness-to-pay threshold of BRL 40,000.00. Pernambuco, 2023 (n=4,092)

Tornado diagrams demonstrated that, after assigning minimum and maximum values to costs and utilities, the most sensitive variables exerting the greatest impact on the incremental cost-effectiveness ratio were: “utility with intrauterine device use without pregnancy” in Scenario 1, which reduced this ratio (Figure 2). 

Conversely, the variables “utility with intrauterine device use with pregnancy” and “cost of Scenario 1” increased the ratio, but they remained below the willingness-to-pay threshold. These findings confirm the cost-effectiveness evaluation, identify Scenario 1 as the most cost-effective, and demonstrate the robustness of the model (Figure 2).

For the dominated scenarios (Scenarios 2 and 3), the behavior of the variables and recalculated values showed an increase in the incremental cost-effectiveness ratio, confirming that these scenarios are not cost-effective in the short term compared with the Pernambuco scenario (Figure 2).

**Figure 3:**
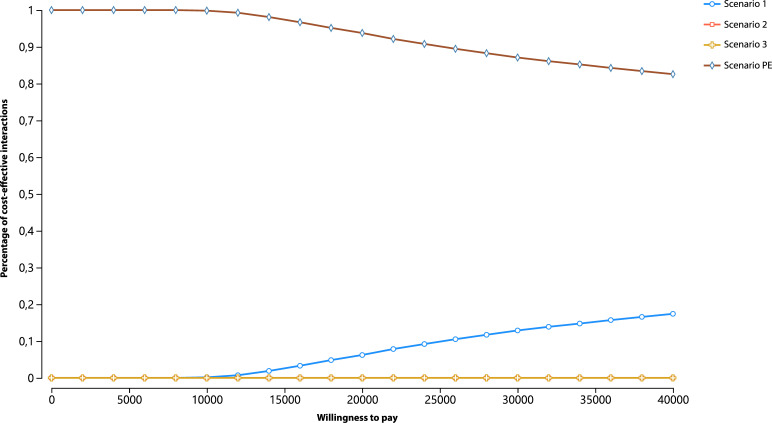
Probabilistic sensitivity analysis. Cost-effectiveness acceptability curve based on the willingness to pay across the four scenarios. Pernambuco, 2023 (n=4,092)

In the probabilistic sensitivity analysis, the cost, utility, probability, relative risk, and discount rate parameters were simultaneously varied using a Monte Carlo simulation with 10 thousand iterations. In each iteration, calculations were performed with different input sets generated from distributions reflecting parameter uncertainty, including mean and standard deviation (Figure 3).

Results were visualized using acceptability curves, which show how input variations affect model outputs, providing a comprehensive view of associated uncertainties. The TreeAge Pro acceptability curve displayed the percentage of cost-effective iterations for each strategy across a range of willingness-to-pay values per QALY gained. Net monetary benefits were calculated for each strategy, and the percentage of iterations in which each achieved the highest net monetary benefit was presented (Figure 3). 

As the willingness-to-pay threshold increased, the acceptability of more effective strategies also increased. Across the 10 thousand iterations, the Pernambuco scenario remained the most effective within the willingness-to-pay limit, with a trend toward greater acceptability for Scenario 1. Scenarios 2 and 3 overlapped in their curve distributions, with no cost-effective iterations observed in the Monte Carlo simulation (Figure 3). 

**Figure 4:**
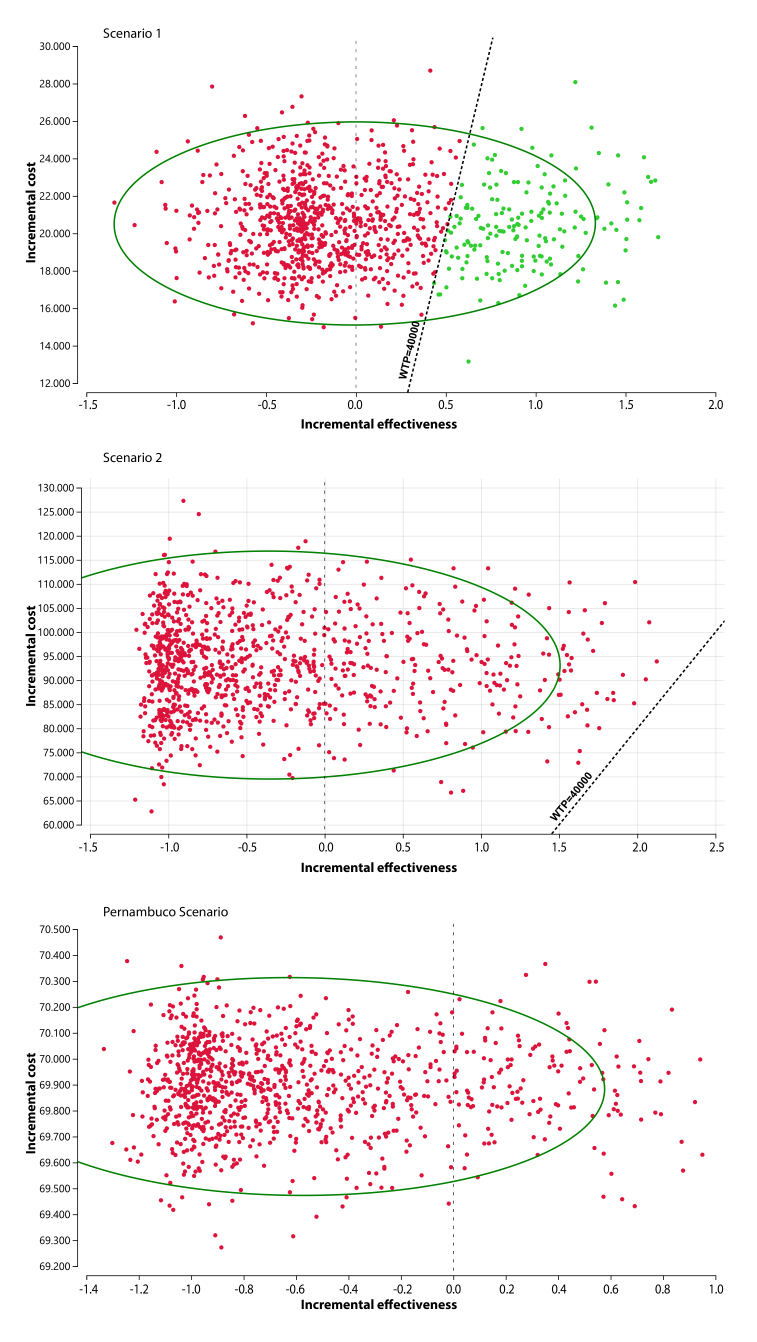
Probabilistic sensitivity analysis. Dispersion of the incremental cost-effectiveness ratio comparing Scenarios 3 and 1 (A), Scenarios 3 and 2 (B), and Scenarios 3 and Pernambuco (C). Pernambuco, 2023 (n=4,092)

Analysis of the incremental cost-effectiveness scatterplot comparing Scenario 3 (reference—highest cost) with scenarios 1, 2, and Pernambuco revealed the relationship between incremental cost and effectiveness of the studied strategies. When comparing Scenario 3 with Scenario 1, Scenario 1 showed a lower cost and a 0.7 reduction in incremental effectiveness (Figure 4). 

Comparing Scenario 3 with Scenario 2, there was a trend of reduced incremental cost with gains in effectiveness, as indicated by the dashed line representing the cost-effectiveness threshold. Points below the line represent simulations in which Scenario 2 is cost-effective. When comparing Scenario 3 with the Pernambuco scenario, Pernambuco was more economical but less effective. The distributions of the iterations remained within the 95% confidence interval, with each point representing a calculation of incremental cost and effectiveness (Figure 4). 

## Discussion 

The findings of this study show that the choice of care strategy impacts both costs and clinical outcomes. 

One of the strategies demonstrated a balance between cost and effectiveness, proving feasible across alternative willingness-to-pay thresholds for incorporating health technologies in Brazil, while also accounting for its innovative nature and its contribution to promoting equity. 

The sensitivity analysis confirmed the robustness of the results. Even with parameter uncertainties, the dominance trend of certain strategies remained consistent.

Some limitations must be considered. The use of data on costs and probabilities derived primarily from national sources and from the Brazilian health system restricts the external validity of the findings to international contexts, especially in the absence of a long-term cohort or a controlled study (19). Underreporting in databases may lead to underestimated figures, thereby affecting the precision of the results. Moreover, due to the model’s complexity and the short follow-up period, side effects of intrauterine devices, discontinuation rates, or expulsions within the first three months were not included. The sustained motivation of trained nurses to continue offering the service could also not be predicted. 

Despite these limitations, the demonstrated feasibility and effectiveness of expanding intrauterine device provision suggest that health managers should consider implementation in decision-making processes to improve population health indicators and optimize the use of both human and financial resources. 

For this adoption to be effective, it is essential to overcome structural challenges that limit its applicability. A clear example is the low rate of intrauterine device insertions in Pernambuco, which remains significantly below the national average of 2% (25). This reflects organizational barriers, overly restrictive clinical criteria, and a shortage of trained professionals, especially nurses. 

Such a scenario highlights the need for strategies that combine professional training, optimization of care workflows, and expansion of access, ensuring that proven effective technologies fulfill their role in public health.

The notion that low adherence to intrauterine device insertion is due to fear or lack of interest among women has been refuted by evidence showing increased informed choice of the method when adequate information on risks and benefits is provided (26,27). 

By removing knowledge and access barriers, preference for this contraceptive method increases due to its duration, effectiveness, and availability, subsidized by the SUS. Therefore, expanding access and providing detailed information have proven to be essential strategies for promoting intrauterine device use (26,27).

This reality reinforces the need to improve resource management, reduce costs, and enhance the quality of health services (28). Expanding provision, coupled with professional training, is an essential strategy to minimize waste and improve the quality of care (29). 

Experiences from health services in Brazil and worldwide have shown that theoretical and practical training of professionals improves counseling, clarifies doubts, and increases adherence to contraceptive methods, contributing to the reduction of unintended pregnancies (29,30).

In Pernambuco, through a cooperation agreement between the Pan American Health Organization (PAHO) and the Women’s Health Care Management Office (*Gerência de Atenção à Saúde da Mulher*, GEASM), training courses are offered to facilitate the enrollment of eligible women on waiting lists and to enhance professional qualifications. In Pernambuco, cost analysis using the absorption costing method showed that the investment required to train nurses is low, despite generating high service value (17). 

An economic evaluation using dynamic modeling demonstrated the superior cost-effectiveness ratio of long-acting reversible contraceptive methods compared with short-acting ones (5). 

Despite its importance, there is still a shortage of studies evaluating the cost-effectiveness of advanced nursing practice in expanding access to intrauterine devices. Integrating this practice with financial efficiency would achieve goals of safety, quality, user satisfaction, and delivery of valuable outcomes at a reasonable cost, addressing the current demand for excellence in healthcare delivery. 

The deterministic and probabilistic sensitivity analyses corroborated the cost-effectiveness findings and demonstrated a low risk of bias when maximum and minimum values were applied to the variables included in the evaluation. 

The present study’s findings are consistent with those of the US Contraceptive CHOICE Project (26) and the Australian Contraceptive ChOice pRoject (ACCORd) (27), which evaluated the cost-effectiveness of expanding the provision of long-acting reversible contraceptive methods. A 10-year analysis revealed that, despite high initial costs, the intervention results in long-term cost savings due to the reduction of unintended pregnancies and associated costs with abortions, prenatal care, and deliveries, as well as other intangible costs.

This approach—training nurses in advanced nursing practice—promotes reproductive justice and expands access and efficiency in health care, providing qualified care and enabling women’s autonomy in reproductive decision-making (30).

The proposed new service delivery strategy demonstrated cost-effectiveness in one of the provision scenarios compared with the current offer, and within this context, the developed Markov model showed that financial savings and improved effectiveness can coexist. 

In summary, incorporating evidence-based strategies, such as nurse training, is crucial to promoting more equitable, efficient, and user-centered healthcare delivery. By leveraging cost-effective, sustainable technologies, health managers can ensure broader access to quality care, thereby improving population health outcomes.

## Data Availability

The database used in this study’s analysis is openly accessible to TreeAge Pro Healthcare 2023 users at: https://1drv.ms/u/c/3c29778fe25348ab/EatIU-KPdykggDzXHAAAAAABQFv8ZTMwdboOKeeWFo60BA?e=cqcsrM, or in the OPEN ICPSR repository at: http://doi.org/10.3886/E226722V1.
