# Supplementary material for: Cost-effectiveness of expanding access to intrauterine device provision by nurses in the public health system: an economic decision analysis using a Markov model, Pernambuco, 2023
Source: Epidemiol Serv Saude. 2026 Feb 23;35:e20240803. doi: 10.1590/S2237-96222025v34e20240803.en (PMC12928549; doi:10.1590/S2237-96222025v34e20240803.en)
Supplement: Translation

# Translation: Custo-efetividade da ampliação da oferta de dispositivos intrauterinos por enfermeiros na rede pública de saúde: análise de decisão econômica por modelo de Markov, Pernambuco, 2023

**DOI:** 10.1590/S2237-96222025v34e20240803.pt

**Authors:** Juliana da Silva Nogueira, Camilla Maria Ferreira de Aquino, Juliana Gonçalves de Araújo, Noemia Teixeira de Siqueira, Umbelina Cravo Teixeira Lagioia, Agostinho de Sousa Machado, Adriana Falangola Benjamin Bezerra

**Affiliations:** 1 Universidade Federal de Pernambuco, Ambulatório de Ginecologia e Obstetrícia, Recife, PE, Brasil; 2 Instituto Federal de Pernambuco, Departamento de Enfermagem, Recife, PE, Brasil; 3 Universidade de Pernambuco, Coordenação do MBA em Gestão Empresarial, Recife, PE, Brasil; 4 University of York, York, NYorks, Inglaterra; 5 Universidade Federal de Pernambuco, Departamento de Ciências Contábeis, Recife, PE, Brasil; 6 Universidade Federal de Pernambuco, Departamento de Ginecologia, Recife, PE, Brasil; 7 Universidade Federal de Pernambuco, Departamento de Saúde Pública, Recife, PE, Brasil

**Keywords:** Enfermeiras e Enfermeiros, Capacitação Profissional, Acesso Efetivo aos Serviços de Saúde, Necessidades e Demandas de Serviços de Saúde, Avaliação em Saúde

## Abstract

**Objetivo:**

Analisar o custo-efetividade do aumento da oferta e da inserção de dispositivos intrauterinos após a capacitação de enfermeiros.

**Métodos:**

Utilizou-se uma análise de decisão econômica com modelo de Markov no software TreeAge Pro Healthcare 2023 para comparar quatro cenários em Pernambuco, considerando a população estimada para 2023. O cenário atual (referência) tem 0,24% de oferta de dispositivos intrauterinos. Os demais cenários projetaram aumentos para 2% (cenário 1), 3,2% (cenário 2) e 4% (cenário 3). Foram analisados os custos com capacitação e com distribuição dos dispositivos intrauterinos, utilizando-se o método de custeio por absorção. As utilidades foram estimadas com base na literatura, adotando-se o limiar brasileiro de disposição a pagar de até R$ 40.000,00, com admissão de flexibilidade até R$ 120.000,00 por ano de vida ajustado pela qualidade.

**Resultados:**

O cenário 1 (2% de oferta) foi o que apresentou a melhor relação de custo-efetividade em comparação ao cenário atual, com um custo incremental de R$ 73.720,20 por ano de vida ajustado pela qualidade, ganhos após três meses da capacitação. Os outros cenários foram dominados, ou seja, houve aumento dos custos e da perda de efetividade, o que os mostrou desvantajosos no curto prazo.

**Conclusão:**

Expandir a oferta para 2% com capacitação de enfermeiros é uma estratégia custo-efetiva em comparação à oferta atual. Além de otimizar os recursos públicos, essa medida pode reduzir a demanda não atendida por contracepção.

**Aspectos éticos**

Esta pesquisa respeitou os princípios éticos, obtendo os seguintes dados de aprovação: :

Comitê de ética em pesquisa: Hospital das Clínicas de Pernambuco

Número do parecer: 6.338.193

Data de aprovação: 2/10/2023

Certificado de apresentação de apreciação ética: 74238023.5.0000.8807

Registro do consentimento livre e esclarecido: Não se aplica.

## Introdução 

O planejamento reprodutivo contribui para a redução de gravidezes indesejadas e está alinhado aos Objetivos de Desenvolvimento Sustentável das Nações Unidas (1). No Brasil, métodos contraceptivos de curta e de longa duração são disponibilizados gratuitamente. Contudo, a adesão aos contraceptivos reversíveis de longa duração é baixa, com prevalência de apenas 2% (2). A população possui maior acesso aos métodos hormonais de curta duração, cuja taxa de falha costuma ser mais alta do que a dos dispositivos intrauterinos. Essa baixa adesão contribui para elevados índices de gravidez não planejada, de abortos e de morbidade materna (3,4).

A elevada taxa de gravidez na adolescência no Brasil gera impactos sociais e econômicos superiores ao custo da ampliação de oferta desses contraceptivos (5). Para melhorar o acesso, não basta apenas a aquisição desses insumos pelo Sistema Único de Saúde, é também necessária a capacitação de profissionais para inseri-los (6). Atualmente, médicos predominam na inserção desses dispositivos, embora, no Nordeste, enfermeiros tenham realizado 47% dos procedimentos, indicando tendência de aumento na participação da enfermagem (7).

Dados do Departamento de Informação e Informática do Sistema Único de Saúde (DATASUS) apontam crescimento na oferta de dispositivos intrauterinos em Pernambuco: de 870 inserções em 2020 para 13.417 em 2022. O uso na rede pública passou de 0,029% em 2020 para 0,24% em 2022. Entretanto, em 2017, 44% dos 24.047 dispositivos distribuídos perderam a validade sem registros de uso, representando prejuízo de R$ 1.011.180,00 (US$ 204.916,30). Em 2024, havia 31.195 unidades, totalizando R$ 2.963.525,00 (US$ 553.164,78), que precisavam ser utilizadas até 2028 para evitar novas perdas. 

Apesar da disponibilidade de insumos para ofertar métodos contraceptivos de longa duração (8), o acesso aos serviços de saúde sexual e reprodutiva no Brasil ainda é particularizado e desigual, pois existe quantidade insuficiente de profissionais, médicos e enfermeiros capacitados para manter a oferta proporcional à demanda (9). Em 2022, a prevalência de gravidez indesejada foi de 62%, com maior incidência entre mulheres jovens, pardas e sem companheiro (2), reforçando a necessidade de estratégias mais eficazes na oferta de métodos contraceptivos. A capacitação de enfermeiros pode ampliar a oferta. 

A Resolução nº 690/2022 do Conselho Federal de Enfermagem estabeleceu diretrizes para a capacitação, permitindo consultas e inserções de dispositivos intrauterinos por enfermeiros (10). Desde 2022, o Conselho Federal e os Conselhos Regionais de Enfermagem iniciaram capacitações e sistematizações de procedimentos nos estados brasileiros com o Projeto de Consulta de Enfermagem Ginecológica, com ênfase no planejamento sexual e reprodutivo (11). A formação adequada permite aos enfermeiros oferecerem educação em saúde, sensibilizar usuárias e fornecer assistência integral de forma segura e eficiente (12,13). A inserção desse tipo de contraceptivo é simples, com baixa incidência de dor intensa e com baixo risco de complicações (14). Experiências internacionais, como no Canadá, mostraram que expandir o escopo de prática dos enfermeiros pode superar barreiras no acesso à saúde reprodutiva (15). 

Considerando-se os potenciais impactos econômicos da ampliação de profissionais habilitados a partir de capacitações, de adaptações da infraestrutura das unidades, de aquisição de insumos e de reorganização da rotina dos serviços de saúde, essa intervenção deve, previamente, ser avaliada quanto ao seu custo-efetividade, conforme recomenda a Comissão Nacional de Incorporação de Tecnologias no Sistema Único de Saúde (16).

Diante dessa possibilidade, este estudo buscou analisar o custo-efetividade do aumento da oferta e da inserção de dispositivos intrauterinos após a capacitação de enfermeiros. Os resultados visam fornecer subsídios para políticas públicas que promovam métodos contraceptivos eficazes, o que acaba por melhorar os indicadores de saúde e econômicos das mulheres brasileiras.

## Métodos 

### Delineamento 

Realizou-se uma avaliação de custo-efetividade sobre a ampliação da oferta de dispositivos intrauterinos por enfermeiros na rede pública de Pernambuco. A elaboração do modelo seguiu o guia *Padrões Consolidados para o Relato de Avaliações Econômicas da Saúde 2022* e foi desenvolvido no software TreeAge Pro Healthcare 2023.

### População-alvo 

As participantes do estudo foram meninas e mulheres sexualmente ativas, de 10 a 49 anos, que não desejavam engravidar, atendidas por enfermeiros após a capacitação.

A amostra de cada cenário foi baseada em dados do Sistema de Informação Ambulatorial - Departamento de Informação e Informática do Sistema Único de Saúde (SIA/SUS-DATASUS) com a consideração do número de inserções de dispositivos intrauterinos em Pernambuco em 2023. O tamanho da amostra foi calculado com base na prevalência do uso de dispositivos intrauterinos nos cenários hipotéticos, com N*0,0024=600 para o cenário Pernambuco (PE), N*0,02=734 para o cenário 1, N*0,032=1.226 para o cenário 2 e N*0,04=1.532 para o cenário 3.

### Contexto e localização

Em Pernambuco, a inserção de dispositivos intrauterinos pode acontecer nos serviços de saúde da atenção primária sob a responsabilidade das gestões municipais, como Centros de Saúde e Policlínicas. Em âmbito estadual, os hospitais regionais contam com ambulatórios de planejamento reprodutivo, no qual as pacientes, reguladas pelas 12 regionais de saúde, podem ser atendidas. Para a simulação, foram utilizados dados de 2023, coletados a partir de sistemas de informação de livre acesso de Pernambuco, com foco em dados sociodemográficos e assistenciais da atenção primária. 

### Perspectiva do estudo

O estudo teve como perspectiva primária o Sistema Único de Saúde (SUS). 

### Comparadores 

A tecnologia em saúde sob estudo foi a inserção de métodos contraceptivos reversíveis de longa duração por enfermeiros da atenção primária após capacitação. Quatro cenários de ampliação de oferta do mesmo método contraceptivo foram avaliados: cenário Pernambuco, o cenário de referência, (probabilidade de oferta atual em Pernambuco, 0,0024); cenário 1 (aumento para 0,02, média nacional); cenário 2 (aumento para 0,032); e cenário 3 (aumento para 0,04).

### Horizonte temporal

Em cada cenário, foi considerado o período de 3 meses após as capacitações. Cada capacitação possui duração mensal (17) e foram adotadas transições mensais entre estados de saúde, pois as participantes poderiam transitar de “não grávida” para “grávida” nesse intervalo. O período de avaliação não foi expandido pela ausência de dados acerca dos efeitos em longo prazo de capacitações sobre a inserção de dispositivos intrauterinos.

### Taxa de desconto

Não foi considerada taxa de desconto, pois o universo temporal de análise for inferior a 1 ano (18).

### Desfechos em saúde

Os anos de vida ajustados por qualidade foram definidos como desfecho primário (19). A efetividade foi medida pela capacidade de produção de resultados desejados, como evitar gravidez, por meio do aumento da oferta de dispositivos intrauterinos entre pessoas elegíveis no âmbito da Estratégia Saúde da Família. As análises estatísticas e de custo-efetividade ocorreram em 2023. 

As medidas de utilidade variaram entre 0 e 1 e foram discriminadas como: a) utilidade com dispositivo intrauterino sem gravidez (no valor de 1); b) utilidade sem dispositivo intrauterino e sem gravidez (no valor 0,8) (20).

Nas situações em que, apesar do uso do dispositivo intrauterino e do desejo de evitar a gravidez, a mulher engravidou, observou-se uma redução na qualidade de vida, na percepção de bem-estar e na confiança no método. Para esse desfecho, foram atribuídos valores de utilidade decremental (desutilidade) à variável “uso de dispositivo intrauterino com ocorrência de gravidez”, estimada em -0,1 (20).

### Limiar de custo-efetividade

O limiar de disposição a pagar foi considerado em R$ 40.000,00 por anos de vida ajustados pela qualidade. No Brasil, as diretrizes de limiares de disposiçãsiao a pagar admitem flexibilidade na adoção desses limiares em situações coerentes com a hipótese de limiares alternativos, por promoverem a inovação e a equidade em saúde para o SUS. Nesses casos, é aceitável um limiar de valores estimados até R$ 120.000,00 (21).

### Custos considerados

Foram incluídos custos diretos das estratégias sob a investigação, incluindo-se: a) dispositivo intrauterino; b) capacitação da equipe em termos individual, baseado no estudo conduzido em Pernambuco (17); c) custo da gravidez indesejada no Brasil, atualizado para 2023 (22); d) custo da consulta individual sem dispositivo intrauterino (tabela do SUS).

### Fontes de dados e mensuração 

As tecnologias avaliadas incluíram a manutenção da oferta atual em Pernambuco (probabilidade de 0,0024), calculada com base em informações coletadas no DATASUS. Os aumentos hipotéticos para 0,02 simularam a probabilidade nacional de uso de dispositivos intrauterinos (2). No segundo cenário hipotético, essa estimativa elevou-se em 60% com a oferta e a inserção de dispositivo intrauterino após a capacitação de profissionais da atenção primária (23), de forma que a probabilidade ficou em 0,032. 

No último cenário hipotético, foi avaliado um aumento de 100% da oferta praticada no território brasileiro, representado pela probabilidade de 0,04 de uso de dispositivos intrauterinos. 

Os desfechos analisados foram gravidez evitada e gravidez indesejada com e sem dispositivos intrauterinos de cobre. O custo da capacitação de enfermeiros foi calculado em R$ 1.820,38 por profissional, com custo unitário de inserção de R$ 91,02 (17). O custo por cenário considerou o custo de inserção do dispositivo multiplicado pela quantidade de usuárias elegíveis em cada cenário. Custos adicionais, como consultas sem dispositivos intrauterinos e sem gravidez indesejada, foram obtidos da tabela do SUS e de estudos brasileiros (22,24) ajustados para 2023 pelo Índice Nacional de Preços ao Consumidor Amplo.

### Modelo de Markov

O modelo foi desenvolvido para simular os efeitos clínicos e econômicos da capacitação de enfermeiros na inserção de dispositivos intrauterinos em uma coorte hipotética de mulheres em idade fértil, elegíveis para o uso de dispositivos intrauterinos, avaliada por três meses após a capacitação. A avaliação de custo-efetividade teve o objetivo de antecipar os custos e os efeitos da capacitação e do aumento da oferta antes da intervenção assistencial pelo gestor local. A tecnologia em saúde foi testada considerando eventos repetitivos (por exemplo: uso ou não do método contraceptivo → gravidez → perda gestacional → novo uso ou não do método), com estados transicionais mensais e dependência temporal das probabilidades e das utilidades associadas, o que permitiu uma representação mais precisa da estrutura clínica avaliada. As pessoas estudadas poderiam transitar entre diferentes estados de saúde: grávida sem dispositivo intrauterino não grávida sem dispositivo intrauterino, não grávida com dispositivo intrauterino e grávida com dispositivo intrauterino.

### Análise de sensibilidade

As análises de sensibilidade foram conduzidas para avaliar o impacto das incertezas nos resultados. Parâmetros de custo e de utilidade foram considerados em análises determinísticas (diagramas de tornado) e probabilísticas (simulação de Monte Carlo com 10 mil interações) com a utilização de dados da literatura. Os parâmetros utilizados estão na Tabela Suplementar 1.

## Resultados 

Na análise de custo-efetividade, comparou-se os quatro cenários ao longo de três meses, o que mostrou que os cenários Pernambuco e 1 não são dominados, enquanto os cenários 2 e 3 o são, ou seja, apresentam custos mais altos sem ganhos de efetividade. 

A ampliação de oferta de dispositivos intrauterinos do cenário Pernambuco para o cenário 1 representou um incremento de 1.233%, calculado pela razão (0,032-0,0024)/0,0024x100.

A razão de custo-efetividade incremental indicou ainda que o cenário 1 está dentro de limiares alternativos de disposição a pagar, no valor de R$ 73.720,20/anos de vida ajustados pela qualidade, quando são admitidos valores até R$ 120.000,00.

Dentro da construção do ranking da avaliação de custo-efetividade, a apreciação dos benefícios monetários líquidos também confirmou o cenário 1 como a melhor opção em termos de custo-efetividade, sendo o mais vantajoso ao considerar a disposição a pagar (Tabela 1). 

**Tabela 1:** Ranking da avaliação de custo-efetividade entre os três cenários de ampliação da oferta de inserção de dispositivos intrauterinos entre mulheres que não desejam engravidar, segundo a razão de custo-efetividade incremental e benefícios monetários líquidos. Pernambuco, 2023 (n=4.092)

Estratégia	Dominância		Custo (R$)	Custo incremental (R$)	Efetividade	Efetividade incremental	Razão de custo efetividade incremental	Benefícios monetários líquidos
Cenário Pernambuco		Não dominado	3.813	-	4,3	-	-	-
Cenário 1		Não dominado	19.655	15.842	4,5	0,2	73.720,20	233.073.733
Cenário 2		dominado	48.003	28.348	4,0	-0,5	-55.273,57	206.376.044
Cenário 3		dominado	73.819	54.165	3,7	-0,7	-73.866,25	194.888.795

Elaborou-se a curva de avaliação de custo-efetividade entre os três cenários hipotéticos, sendo o cenário 1 o que apresenta o aumento incremental de efeito (anos de vida ajustados para qualidade ganhos) com menor custo ao ser comparado aos demais cenários alternativos (Figura 1). 

**Figura 1:** Análise de custo-efetividade da comparação dos quatro cenários de oferta de dispositivos intrauterinos. Pernambuco, 2023 (n=4.092)

Nas análises de sensibilidade, investigou-se a incerteza nos parâmetros do modelo, recalculando valores de custos e de utilidades (análise determinística). 

Os diagramas de tornado evidenciam que, após atribuir valores máximos e mínimos para os custos e as utilidades, as variáveis mais sensíveis e que exercem maior impacto na razão de custo-efetividade incremental são: “utilidade com dispositivo intrauterino e sem gravidez”, no cenário 1, com redução dessa relação (Figura 2). 

**Figura 2:** Análise de sensibilidade determinística. Variação na razão de custo-efetividade incremental, quando comparados cenários alternativos (A, B e C) versus cenário Pernambuco, com disposição a pagar: R$ 40.000,00. Pernambuco, 2023 (n=4.092)

Já as variáveis “utilidade com dispositivo intrauterino e com gravidez” e “custo do cenário 1” impactam no aumento da relação, mas se mantêm abaixo da disposição a pagar. Esses achados confirmam a avaliação de custo-efetividade com o cenário 1 mais custo-efetivo e evidenciam a robustez do modelo (Figura 2).

Nos demais cenários, os que foram dominados (cenários 2 e 3), observou-se que o comportamento das variáveis empregadas e dos recálculos evidencia aumento da razão de custo-efetividade incremental, o que confirma não serem relações custo-efetivas em curto prazo, quando comparadas ao cenário Pernambuco (Figura 2).

Na análise de sensibilidade probabilística, as variáveis de custos, de utilidades, de probabilidades, de risco relativo e de taxa de desconto foram simultaneamente alteradas, com uso da simulação de Monte Carlo com 10 mil interações. Em cada interação, os cálculos foram realizados com diferentes conjuntos de entradas, gerados a partir de distribuições que refletiram a incerteza dos parâmetros, incluindo média e desvio padrão (Figura 3).

**Figura 3:** Análise de sensibilidade probabilística. Curva de aceitabilidade de custo-efetividade, pela disposição a pagar entre os quatro cenários. Pernambuco, 2023 (n=4.092)

Para visualizar os resultados, foram utilizadas curvas de aceitabilidade, que demonstram como as variações nas entradas impactam as saídas do modelo, o que oferece uma visão abrangente das incertezas associadas. A curva de aceitabilidade do TreeAge Pro mostrou a porcentagem de interações custo-efetivas de cada estratégia dentro de um intervalo de disposição a pagar por anos de vida ajustados para qualidade. Foram calculados os benefícios monetários líquidos para cada estratégia e apresentada a porcentagem de cálculos em que cada uma teve o maior benefício monetário líquido (Figura 3). 

À medida que a disposição a pagar aumenta, se eleva também a aceitabilidade das estratégias mais eficazes. Ao longo das 10 mil interações, o cenário Pernambuco se manteve como o mais eficaz dentro do limite de disposição a pagar, com uma tendência de aumento na aceitabilidade do cenário 1. Os cenários 2 e 3 sobrepuseram-se na distribuição da curva, com ausência de interações custo-efetivas na simulação de Monte Carlo (Figura 3). 

A análise do gráfico de dispersão da razão de custo-efetividade incremental entre o cenário 3 (referência - com maior custo) e os cenários 1, 2 e Pernambuco revelou a relação entre custo e efetividade incremental das estratégias estudadas. Comparando o cenário 3 com o cenário 1, o cenário 1 apresentou menor custo, além de redução de efetividade incremental de 0,7 (Figura 4). 

**Figura 4:** Análise de sensibilidade probabilística. Dispersão da razão de custo-efetividade incremental, comparando-se os cenários 3 e 1 (A), cenários 3 e 2 (B) e cenários 3 e Pernambuco (C). Pernambuco, 2023 (n=4.092)

Na comparação do cenário 3 com o cenário 2, houve tendência de redução do custo incremental com ganhos em efetividade, no que a linha pontilhada indica o limiar de custo-efetividade. Os pontos abaixo da linha representam simulações nas quais o cenário 2 é custo-efetivo. Ao comparar o cenário 3 com o cenário Pernambuco, o cenário Pernambuco mostrou ser mais econômico, embora menos efetivo do que o cenário 3. As distribuições das interações se mantiveram dentro do Intervalo de Confiança de 95%, com cada ponto representando um cálculo de custo e de eficácia incremental (Figura 4). 

## Discussão 

Os achados deste estudo mostram que a escolha da estratégia de cuidado impacta custos e desfechos clínicos. 

Uma das estratégias apresentou um equilíbrio entre custo e efetividade, viável dentro de limiares alternativos de disposição a pagar adotados para a incorporação de tecnologias em saúde no Brasil, com a consideração do caráter inovador da estratégia e da promoção de equidade. 

A análise de sensibilidade confirmou a robustez dos resultados. Mesmo com incertezas nos parâmetros, a tendência de dominância de certas estratégias permaneceu.

Algumas limitações devem ser consideradas. A utilização de dados sobre custos e probabilidades provenientes majoritariamente de fontes nacionais e do sistema de saúde brasileiro restringe a generalização dos achados para contextos internacionais, especialmente na ausência de uma coorte de longo prazo ou de um estudo controlado (19). O sub-registro nas bases de dados pode levar a estimativas subdimensionadas, o que impacta a precisão dos resultados. Além disso, devido à complexidade do modelo e ao curto período de acompanhamento, não foram incluídos efeitos colaterais dos dispositivos intrauterinos, taxa de descontinuação ou expulsão nos primeiros três meses. A permanência da motivação dos enfermeiros capacitados para manter a oferta do serviço também não pôde ser prevista. 

Apesar dessas limitações, a viabilidade e a efetividade comprovadas da ampliação de oferta dos dispositivos sugeriram que a implementação deve ser considerada pelos gestores na tomada de decisão, com vistas à melhoria dos indicadores de saúde da população e à utilização eficiente dos recursos humanos e dos financeiros disponíveis. 

Para que essa adoção seja eficaz, é essencial superar desafios estruturais que limitam sua aplicabilidade. Um exemplo evidente é a baixa taxa de inserção de dispositivos intrauterinos em Pernambuco, que se mostra significativamente inferior à média nacional, com média de 2% (25). Isso reflete barreiras organizacionais, critérios clínicos excessivos e escassez de profissionais capacitados, especialmente enfermeiros. 

Tal cenário ressalta a necessidade de estratégias que combinem capacitação profissional, otimização dos fluxos assistenciais e ampliação do acesso, o que garante que tecnologias comprovadamente efetivas cumpram seu papel na saúde pública.

A alegação de que a baixa adesão na colocação de dispositivos intrauterinos se deva ao medo ou ao desinteresse das mulheres foi refutada com a demonstração de aumento da escolha consciente pelo método quando existe acesso a informações adequadas sobre riscos e benefícios (26,27). 

Ao eliminar barreiras de conhecimento e de acesso, a preferência por essa contracepção cresce em decorrência da sua duração, da sua eficácia e da sua disponibilidade, subsidiada pelo SUS. Assim, ampliar o acesso e fornecer informações detalhadas se mostraram estratégias essenciais para promover a utilização desses dispositivos (26,27).

Essa realidade reforça a necessidade de melhorar a gestão de recursos, de reduzir os custos e de otimizar a qualidade dos serviços de saúde (28). A ampliação da oferta, aliada à capacitação profissional, é estratégia essencial para mitigar o desperdício e qualificar a assistência (29). 

Experiências em serviços de saúde no Brasil e no mundo demonstraram que os treinamentos teórico e prático dos profissionais melhoram o aconselhamento, o esclarecimento de dúvidas e as taxas de adesão aos contraceptivos, contribuindo para a redução da gravidez indesejada (29,30).

Em Pernambuco, por meio de termo de cooperação entre a Organização Pan-Americana da Saúde e a Gerência de Atenção à Saúde da Mulher, são promovidos cursos de capacitação, o que viabiliza a convocação de usuárias aptas em fila de espera e a qualificação de profissionais. Em Pernambuco, a análise do custeio por absorção evidenciou que o investimento necessário para capacitar enfermeiros é reduzido, apesar de gerar elevado valor assistencial (17). 

Em avaliação econômica com modelagem dinâmica, foi evidenciada a superioridade da relação de custo-efetividade dos métodos contraceptivos de longa duração sobre os de curta duração (5). 

Apesar da importância, ainda há escassez de estudos que avaliem custo-efetividade da enfermagem de prática avançada em prol da ampliação da oferta dos dispositivos intrauterinos. Integrar essa prática com eficiência financeira acarretaria metas de segurança, de qualidade, de satisfação da usuária e de entrega de resultados valiosos a um custo razoável, o que atenderia à demanda atual por excelência assistencial. 

As análises de sensibilidade determinística e probabilística corroboraram os achados da avaliação de custo-efetividade e evidenciaram que existe baixo risco de viés quando realizadas alterações de valores máximos e mínimos atribuídos às variáveis que entraram na avaliação. 

Os achados do estudo em pauta convergem com os estudos US Contraceptive Choice Project (CHOICE) (26) e Australian Contraceptive ChOice pRoject (ACCORd) (27), que avaliaram a relação de custo-efetividade do aumento da oferta de métodos contraceptivos reversíveis de longa duração. A análise de 10 anos revelou que, apesar dos altos custos iniciais, a intervenção resulta em economia de recursos ao longo do tempo, devido à redução de gravidezes indesejadas e dos custos associados a abortos, a cuidados pré-natais e a partos, além de a outros custos intangíveis.

Essa abordagem, que se baseia na capacitação de enfermeiros em prática avançada de enfermagem, não apenas promove justiça reprodutiva, mas também amplia o acesso e a eficiência dos serviços de saúde, o que fornece cuidados qualificados e facilita o livre-arbítrio na tomada de decisão sobre a gravidez entre interessadas no método (30).

A proposta da nova estratégia de oferta demonstrou a relação custo-efetividade em um dos cenários de oferta, quando comparado com a oferta atual, e nesse contexto, o modelo de Markov desenvolvido mostrou que economia de recursos financeiros e melhoria de efetividade podem coexistir. 

Em suma, a incorporação de estratégias baseadas em evidências, como a capacitação de enfermeiros, é fundamental para promover uma prestação de serviços de saúde mais equitativa, eficiente e centrada nas usuárias de serviços de saúde. Ao aproveitar tecnologias custo-efetivas e sustentáveis, os gestores de saúde podem garantir um acesso mais amplo a cuidados de qualidade, o que contribui para o alcance de melhores resultados de saúde.

## Data Availability

O banco de dados utilizado na análise da pesquisa está disponível para livre acesso entre usuários do TreeAge Pro Healthcare 2023 no endereço: https://1drv.ms/u/c/3c29778fe25348ab/EatIU-KPdykggDzXHAAAAAABQFv8ZTMwdboOKeeWFo60BA?e=cqcsrM ou no repositório OPEN ICPSR no endereço: http://doi.org/10.3886/E226722V1.
